# Winter Wheat Water Productivity Evaluated by the Developed Remote Sensing Evapotranspiration Model in Hebei Plain, China

**DOI:** 10.1155/2015/384086

**Published:** 2015-01-06

**Authors:** Shengwei Zhang, Hongbin Zhao, Huimin Lei, Hongbo Shao, Tingxi Liu

**Affiliations:** ^1^Water Conservancy and Civil Engineering College, Inner Mongolia Agricultural University, Hohhot 010018, China; ^2^College of Life Sciences, Inner Mongolia Agricultural University, Hohhot 010018, China; ^3^State Key Laboratory of Hydroscience and Engineering, Department of Hydraulic Engineering, Tsinghua University, Beijing 100084, China; ^4^Key Laboratory of Coastal Biology & Bioresources Utilization, Yantai Institute of Coastal Zone Research (YIC), Chinese Academy of Sciences (CAS), Yantai 264003, China; ^5^Institute of Biotechnology, Jiangsu Academy of Agricultural Sciences, Nanjing 210014, China

## Abstract

Agricultural water is the main reason for the rapid decline of the NCP groundwater levels. It is of vital importance for the NCP sustainable agricultural development to master the ETa and its CWP. In this paper, the EBEM model was developed according to the theory of energy balance. From 2001 to 2006, the winter wheat ETa and CWP were estimated, and the spatial and temporal variations and their influencing factors were studied in the Hebei Plain. The results indicate that the EBEM model performed well by applying MODIS data to estimate the daily net radiation and ETa. For the daytime net radiation, the relative error between the estimation and the measurement amounted to 8.2% and the SEE was 0.82 MJ m^−2^/day. The average ETa deviation between the estimates and the measures amounted to 0.86 mm daily, and the SEE was 1.2 mm. The spatial variations indicated that the major distribution of ETa ranged from 350 to 450 mm, which trended downward within the study area from west to east. In the study period, the winter wheat CWP was mainly distributed between 0.29 and 1.67 kg/m^3^. In space, the CWP was higher in the west than in the east.

## 1. Introduction

With the worldwide population growth and economic development, the global water demand and the degree of water stress are both increasing. Thus, determining how to acquire a better management of water resources and to achieve sustainable development becomes an important issue that every country's government must consider and solve. Nearly a decade ago, 87% of global water extraction (extraction minus return) was consumed by irrigation. Meanwhile, worldwide food production of those irrigated fields accounted for 40–45% [1]. Therefore, reasonable estimates and scientific management of irrigation water are not only important for the sustainable use of water resources but also related to the issue of food security [2]. However, the scientific management of irrigation depends on the accurate estimation of both crop water productivity (CWP) and crop water consumption (CWC) and can be expressed as active evapotranspiration (ETa) [3, 4]. In the regional perspective, remote sensing refers to one of the suitable means for reasonable estimation of the ETa. In the past few decades, remote sensing-based regional ETa estimation has been used to plan and manage water irrigation with high accuracy and precision [5, 6]. Among all of the remote sensing data being used to estimate ETa, the Moderate Resolution Imaging Spectroradiometer (MODIS) is more suitable for medium-to-large regional-scale application because of its advantage of high time resolution, which means medium spatial resolution, high spectral resolution, and free access to the information [7–9], even in a data scarce and heterogeneous landscape [10].

The North China Plain (NCP), an area of 320,000 km^2^ with a population of over 300 million, is one of the most important grain-producing areas in China. The arable land area of the NCP amounts to 1.2 million hectares, accounting for 18.3% of the national total, and its food production provides 21.6% of China's national total. In this region, the main planting agency is the winter wheat-summer corn rotation each year, while the growing season of the winter wheat is from early October to the end of May in the following year. While the NCP is in the monsoon climate with very little rainfall, the multiyear average rainfall (1960–2005) is only 119 mm. The amount of precipitation is even smaller than one-third of the requirement for achieving the average yield [11].

Therefore, irrigation is necessary to ensure adequate food production. Generally, there are three to five time periods of irrigation for a total of 300–400 mm of water per year. As a result, irrigation water accounts for approximately 70% of the total water consumption of the local area, while winter wheat consumes almost 70% of the total amount of irrigation water. Therefore, approximately 50% of the water is consumed by winter wheat [12].

The relationship between ETa and CWP was primarily studied by adjusting the irrigation methods and the irrigation amount in the NCP [13, 14]. Although these experiments achieved meaningful results, the scale of the field experiments cannot reflect the spatial variability and is not sufficiently representative. The GIS-based model was also used to research the CWP variation in the NCP [15]. However, the crop water consumption in that research study was obtained by indirect calculation of electricity consumption. Mo et al. simulated the ETa and CWP temporal and spatial variations in the SVAT model of the NCP. This model requires a number of input data, such as DEM, soil texture, and meteorological data, and, thus, the additional required data and parameters are very inconvenient for utilization of the model [16]. Li et al. estimated the distribution of the ETa and CWP by applying AVHRR data input to the SEBAL model in the year of 2003 [12]. However, the model did not adequately describe either the space representation or the interannual variation. Tian et al. estimated the ETa from 2001 to 2009 but the CWP was not considered [17]. Thus, it is necessary to perform further study on the temporal variation and the spatial distribution of ETa and CWP to provide a reference for agricultural water management and sustainable regional development.

Although the statistical data at the spatial resolution is less than the yield estimation based on the remote sensing data through the model to determine production, the county scale represents the largest scale of production data available in the NCP [15] and is also the basic unit of the agricultural water use and the water resources management. Therefore, in this research, the county scale was selected, and the ETa and CWP of winter wheat were estimated based on the remote sensing data in the NCP. The ETa of the winter wheat planting area was estimated using an energy balance-ET model called the energy balance and evapotranspiration model (EBEM). Through the application of the remote sensing data, the winter wheat areas were classified to determine the counties' average ETa, and the CWP was defined as the ETa divided by the yield. Therefore, the factors influencing the CWP and ETa of winter wheat were analyzed and their temporal and spatial variations were investigated.

## 2. Materials and Methods

### 2.1. Study Area

**Figure 1:**
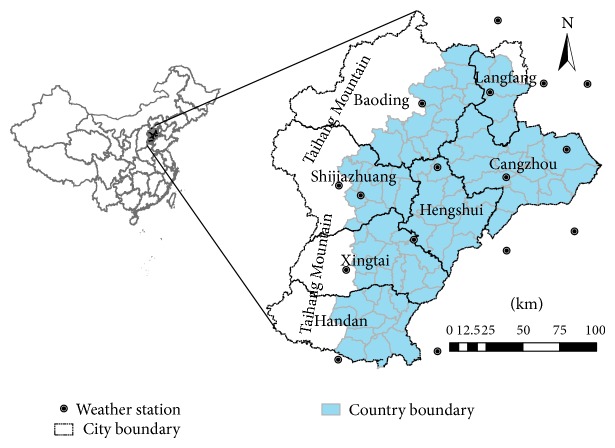
Study area and location of meteorology stations.

In this study, the Hebei Plain (HP), located in the northern part of the NCP, was chosen to represent the NCP. A total of 80 counties with an elevation of below 100 m were included in this area (Figure 1). The local climate is a typical temperate semiarid one with a strong summer monsoon season during a hot and rainy summer and a dry and cold winter. The average monthly temperatures range from −2.5°C to 26°C, and the average annual rainfall is approximately 400 to 500 mm, with approximately 70% of this rainfall occurring from July to September during the monsoon season. The major crop types are the double-crop system of winter wheat and summer maize.

### 2.2. Data

#### 2.2.1. Remote Sensing Data

In this paper, the remote sensing data included four types of MODIS products: MOD08_E3 (Level-3 MODIS Atmosphere Daily Global Product, 1 degree by 1 degree, 8 days) used to obtain the water vapor content of the atmosphere, MOD09A1 (Surface Reflectance Bands 1–7, 500 m, 8 days) used for surface albedo estimates, MOD11A2 (Land Surface Temperature and Emissivity, 1000 m, 8 days) used for extracting the surface temperature and longwave radiation estimates, and MCD43B1 (BRDF-Albedo Model Parameters, 1000 m, 16 days) for the bidirectional reflectance distribution function (BRDF) correction data once every 8 days by the time interpolation. The data were obtained through the EOS Data Gateway from 2001 to 2006. Regarding the scope of this study, a total of four groups of data were required to cover the entire area, namely, “h26v04,” “h26v05,” “h27v04,” and “h27v05.” Such data were processed by splicing, projection conversion, and denoising through the use of the quality control data and the BRDF correction using the parameters of the BRDF.

#### 2.2.2. Meteorological and Ground Observation Data

In this paper, the meteorological data include the following values: daily maximum and minimum temperatures (°C), wind speed (m s^−1^), relative humidity (%), and number of sunshine hours (h), which are obtained from the China Meteorological Data Sharing System (http://cdc.cma.gov.cn/), with the site distribution shown in Figure 1. The data were interpolated to the entire study area by using the Kriging interpolation methods. In addition, the data provided by the radiation observing system and large weighing Lysimeter (Lysimeter) of the Luancheng Agro-Ecosystem Experimental Station of the Chinese Academy of Sciences (Luancheng Station) were provided for the model in this paper. The Lysimeter, with an area of 1.5 m × 2 m, a depth of 2.5 m, an empty weight of two tons and a weight of 14 tons when filled with soil, has an accuracy of up to 0.02 mm [18] where the crops, irrigation, and fertilization are the same as the surrounding farmland. In this region, the main crop type is the winter wheat-summer corn rotation each year.

In this research, the production statistics, including the acreage and winter wheat production from 2000 to 2006 (Hebei Provincial People's Government Office and the Bureau of Hebei Province, 2001–2007), were obtained from the Hebei Rural Statistical Yearbook.

### 2.3. EBEM Theory

The EBEM calculates the latent heat flux by taking advantage of the energy balance principle, as shown in the following equation:

(1) $$\begin{array}{c} \text{LE}=R_{n}-G-H, \end{array}$$

where LE represents the latent heat flux, *R*_*n*_ refers to net radiation reaching the earth's surface, *G* represents the soil heat flux, and *H* refers to the sensible heat flux, with the units in W m^−2^.

#### 2.3.1. Instantaneous Net Radiation

Latent heat flux can be obtained by determining each component of the right-hand side of (1). However, we can only obtain the instantaneous surface information using remote sensing data, as shown in the following equation:

(2) $$\begin{array}{c} R_{n}=\left(1-\alpha \right)R_{s↓}+R_{L↓}-{R_{L}}_{↑}-\left(1-\varepsilon_{o}\right)R_{L↓}, \end{array}$$

where *R*_*s*↓_ represents the incident shortwave radiation, *α* refers to the surface albedo, *R*_*L*↓_ represents the incident longwave radiation, *R*_*L*__↑_ refers to the outgoing longwave radiation, and *ε*_*o*_ denotes the wide band surface thermal emission rate.

The shortwave radiation reaching the surface is calculated as follows:

(3) $$\begin{array}{c} R_{s↓}=\frac{G_{\text{sc}}\cdot \cos\theta_{\mathrm{rel}}\cdot \tau_{\text{sw}}}{d^{2}}, \end{array}$$

where *G*_sc_ represents the solar constant (1367 W m^−2^), *θ*_*rel*⁡_ refers to the sun incident angle, *τ*_sw_ represents the atmospheric transmittance, and *d* ^2^ refers to an average distance squared for one day [19].

The atmospheric transmittance *τ*_sw_ is obtained using the air pressure *P* (kPa), *W* (mm) of the water vapor content in the atmosphere, the solar elevation angle, and turbidity coefficient *K*, as shown in (4), where 0 ≤ *K*_*t*_ ≤ 1.0; when air quality is better, *K*_*t*_ is equal to 1; and when the air is extremely turbid or seriously polluted, *K*_*t*_ is equal to 0.5 [19]:

(4) $$\begin{array}{c} \tau_{\text{sw}}=0.35+0.627\exp\left[\frac{-0.00146P}{K_{t}\cos\theta_{\text{hor}}}-0.075{\left(\frac{W}{\cos\theta_{\text{hor}}}\right)}^{0.4}\right]. \end{array}$$

Allen et al. [19] calculated *W* by using the near-surface water vapor pressure. However, acquiring the near-surface water vapor pressure was limited to the ground station. Therefore, we obtained the water vapor pressure by using the MOD08_E3 of MODIS.

The surface albedo *α* was calculated using the narrowband reflectance estimation method, as shown in the following equation:

(5) α=0.16ρ1+0.291ρ2+0.243ρ3+0.116ρ4+0.112ρ5+0.081ρ7−0.0015,

where *ρ*_*i*_ refers to each band narrowband reflectance [20].

The outing longwave radiation was calculated using (6), where *ε*_*o*_ represents the surface thermal emission rate and *T*_*s*_(*K*) refers to the surface temperature. Both *ε*_*o*_ and *T*_*s*_ are obtained by the MOD11 product, and *σ* is the Stefan-Boltzmann constant (5.67 ∗ 10^−8^ W m^−2 ^K^−4^):

(6) $$\begin{array}{c} R_{Li↑}=\varepsilon_{o}\sigma T_{s}^{4}. \end{array}$$

The incident longwave radiation was calculated using (7), where *ε*_*a*_ is the air thermal emission rate, which can be obtained using (8). *T*_*a*_ refers to the air temperature:

(7) $$\begin{array}{c} R_{Li↓}=\varepsilon_{a}\sigma T_{a}^{4}, \end{array}$$

(8) $$\begin{array}{c} \varepsilon_{a}=0.85{\left(-\ln\tau_{\text{sw}}\right)}^{0.09}. \end{array}$$

**Figure 2:**
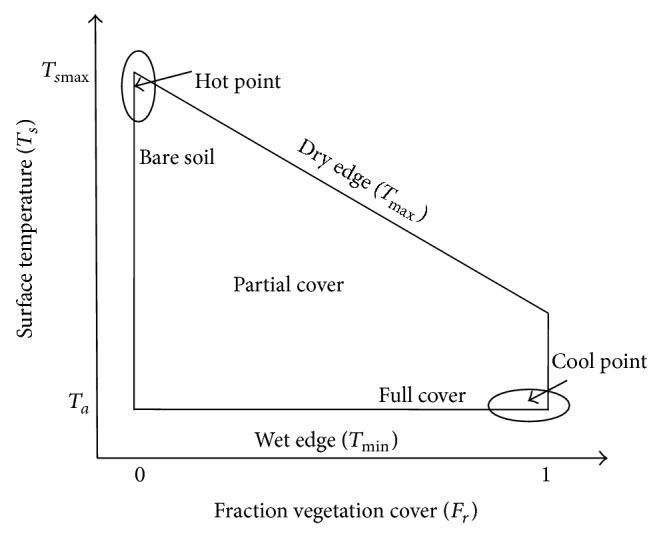
The schematic diagram of the conceptual surface temperature-vegetation fraction (*T*_*s*_-*F*_*r*_) triangular space.

Air temperature can be obtained by the surface temperature of the vegetation coverage concept space, as shown in Figure 2. The space evolved by the surface temperature-vegetation index (*T*_*s*_-VI) space vegetation fraction (*F*_*r*_) can better reflect the relationship between vegetation and soil [21] from the pixel scale because the NDVI or the EVI only represent the green vegetation distribution of the surface.

#### 2.3.2. Calculating the Instantaneous Soil Heat Flux and the Sensible Heat Flux

After obtaining the instantaneous *R*_*n*_, both *G* and *H* must be obtained. To achieve instantaneous LE, *G* was obtained by taking advantage of its relationship with the *R*_*n*_ ratio with the other surface parameters. By using the Luancheng Station observation data and the remote sensing observations of surface temperature, *G* was calculated according to the proposed equation

(9) GRn=−0.319Ts−273.152 +8.575Ts−273.15−53.06Ts−273.15210−3,

where *T*_*s*_(*K*) refers to the surface temperature of the MODIS observations.

The transient thermal flux *H* and the estimation of the relevant parameters, using the procedures of Bastiaanssen et al. [22] and Allen et al. [19], are obtained using an iterative process involving the aerodynamic equation and the Monin-Obukhov similarity theory. *H* was calculated according to the following equation:

(10) $$\begin{array}{c} H=\rho_{\text{air}}C_{p}\frac{dT}{r_{\text{ah}}}, \end{array}$$

where *ρ*_air_ represents the air density (kg m^−3^); *C*_*p*_ refers to the specific heat of air (J kg^−1 ^K^−1^); *r*_ah_ represents the aerodynamic drag (s m^−1^) near the ground between the heights *z*_1_ and *z*_2_; *z*_1_ represents 0.1 m and *z*_2_ refers to 2 m; and *dT* denotes the temperature gradient between *z*_1_ and *z*_2_. Bastiaanssen et al. have proposed the use of a linear relationship between *dT*and *T*_*s*_, as described in the following equation, to calculate *dT* [22]:

(11) $$\begin{array}{c} dT=aT_{s}+b, \end{array}$$

where *a* and *b* refer to empirical parameters, which can be obtained by estimating the area under each ETa regression of the remote sensing images, which can be obtained from at least two points (hot and cold spots). Under normal circumstances, by artificially selecting good vegetation growth, the adequate water supply point is selected as the cold spot, while the area without vegetation and relatively dry is selected as the hot spot. However, such a method adds much uncertainty. In this paper, to avoid this uncertainty, the *T*_*s*_-*F*_*r*_ space automatically obtains the cold and hot spots, which can make the model more appropriate for automated batch computing. As shown in Figure 2, the hot point defined as *F*_*r*_ is very small (close to 0) and has a high surface temperature, while *F*_*r*_ is close to 1 and the surface temperature is the lowest pixel point, which is defined as the cold spot.

#### 2.3.3. Whole Day ETa

Because the value of all-day evapotranspiration is more meaningful than the instantaneous value [19], the daily ET (ET_day_) value was obtained using (12)–(14):

(12) $$\begin{array}{c} {\text{ET}}_{\text{day}}=\frac{86400\text{EF}\left(R_{n\text{day}}-G_{\text{day}}\right)}{\lambda }, \end{array}$$

where EF represents the evaporation ratio and *λ* (J kg^−1^) refers to the latent heat of water vaporization at the specific temperature, which can be obtained from the following equations, respectively:

(13) $$\begin{array}{c} \text{EF}=\frac{\text{LE}}{R_{n}-G}, \end{array}$$

(14) $$\begin{array}{c} \lambda =\left[2.501-0.00236\left(T_{s}-237.15\right)\right]\times 10^{6}. \end{array}$$

Samani et al. assumed that the instantaneous net radiation (*R*_*n*_) and the daytime net radiation (positive net radiation *R*_*n*day_) with instantaneous shortwave radiation (*R*_*s*_) are proportional to the daily shortwave radiation (*R*_*s*_24__) [23] and proposed the following equation to calculate the all-day net radiation *R*_*n*day_:

(15) $$\begin{array}{c} R_{n\text{day}}=R_{n}\frac{R_{s}}{R_{s\text{day}}}{\left(\frac{{T_{a}}_{\text{day}}}{T_{a}}\right)}^{4}, \end{array}$$

where *T*_*a*__day_(*K*) represents the average temperature for the entire day; *T*_*a*_(*K*) denotes the instantaneous temperature; *R*_*s*_ refers to the instantaneous shortwave radiation by using (3); and *R*_*s*day_ represents the shortwave radiation for the entire day by the following equation:

(16) $$\begin{array}{c} R_{s\text{day}}=k_{Rs}{\left(T_{\max}-T_{\min}\right)}^{0.5}R_{a}, \end{array}$$

where *T*_max⁡_ represents the maximum temperature (°C), *T*_min⁡_ represents the minimum temperature (°C), and *k*_*Rs*_ refers to the adjustment factor. For the large coastal areas of air masses approaching water influence, *k*_*Rs*_ was set to 0.19. For the air mass in the inland areas, which are relatively less affected by water, *k*_*Rs*_was set to 0.16 [24]. *R*_*a*_ refers to the atmospheric outer radiation (extraterrestrial radiation) in the following equation:

(17) Ra=1440π×Gscdrϖssinϕsinδ+cos⁡ϕcos⁡δsinϖs,

where *G*_sc_ is the solar constant (0.0820 MJ m^−2 ^min^−1^); *d*_*r*_ denotes an average distance countdown for a day; *ϖ*_*s*_ refers to the sunset hour angle (rad); *φ* marks the latitude (rad); and *δ* represents the real declination (rad).

Given the fluctuations in soil heat flux (*G*_day_) within a full day, Bastiaanssen et al. considered that it can be ignored [22]:

(18) $$\begin{array}{c} G_{\text{day}}=0.122R_{n\text{day}}-0.781. \end{array}$$

#### 2.3.4. ETa Calculation in a Certain Period

The seasonal or annual total ETa is significant for irrigation and water resources management, which can be calculated using the following equation:

(19) $$\begin{array}{c} {\text{ET}}_{\text{period}}=\overset{M}{\underset{i=m}{\sum }}\left[\left({\text{ET}}_{r}F\right)*\left({\text{ET}}_{r\text{day}i}\right)\right], \end{array}$$

where ET_period_ refers to the cumulative evapotranspiration in a certain period, which may be from the beginning of one day to the end of the other day. ET_*r*_ *F* represents the transpiration ratio for this period; ET_*r*day*i*_ denotes the daily reference evapotranspiration, which can be calculated using the meteorological data. The data input in the remote sensing model represents a synthetic product of 8 days and represents the best weather conditions of the 8 days. Therefore, ET_*r*_ *F* was calculated according to the daily evapotranspiration obtained using the remote sensing data and the highest reference evapotranspiration in this period, as shown in the following equation:

(20) $$\begin{array}{c} {\text{ET}}_{r}F=\frac{{\text{ET}}_{\text{day}}}{{\text{ET}}_{r\_\max\_\text{period}}}, \end{array}$$

where ET_*r*_max⁡⁡_period_ refers to the maximum reference evapotranspiration of the synthesis period of 8 days.

### 2.4. ETa Calculation at the County Level

Because there are also many other crops, natural vegetation, and nonvegetation in the study area, a classification method based on time series NDVI data was used for determining the actual winter wheat area so that the water consumption by winter wheat can be accurately estimated [25].

### 2.5. CWP Calculation

In the study area, the crop water productivity of the winter wheat was calculated according to [26]

(21) $$\begin{array}{c} \text{CWP}=\frac{\text{GY}}{{\text{ET}}_{Y}}, \end{array}$$

where CWP (kg m^−3^) refers to the crop water productivity; GY (kg ha^−1^) represents the crop yield; and ET_*Y*_ (mm) denotes the sum of the entire growing season of ETa from October 1 to June 15 in the following year.

## 3. Results and Discussion

### 3.1. EBEM Verification

#### 3.1.1. Verification of Daily Net Radiation

LE refers to the energy, as described by the energy balance equation, which constitutes the source of energy occurring during evapotranspiration. Accordingly, it is very important to accurately estimate the daily net radiation (*R*_*n*day_) for obtaining accurate LE values. In this paper, based on (15), the all-day net radiation was calculated by the instantaneous net radiation. The method was proposed by [23] and verified. However, the method has differences among the study area, vegetation type, and the focus of the research, and it is necessary to evaluate the applicability of the method in the region.

**Figure 3:**
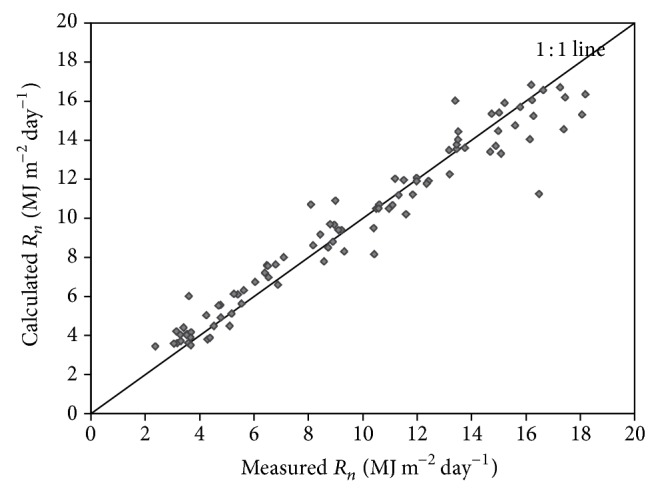
The observed and estimated *R*_*n*_ values in 2005 and 2006.

In Figure 3, the 91 observed data ranges from 2.37 to 18.19 MJ m^−2^ day^−1^ were derived from the Luancheng Station and compared with the estimated value based on the remote sensing data. The average ratio was 0.976, the SEE amounted to 0.82 MJ m^−2^ day^−1^, and the relative error was 6.7%, which was between the estimates and the measured values.

#### 3.1.2. ETa Validation

To evaluate the ETa using the models, the observed evapotranspiration value, which was obtained by the Lysimeter located in the Luancheng Station, was compared with the ETa calculated using the MODIS data. The ground observation data have been called into question because of the differences among the wide ranges of the existing space. However, in previous studies, the data derived from the Luancheng Station Lysimeter have been repeatedly successfully applied to the remote sensing data to estimate the ET verification work (see [12, 27]). Therefore, we also used the previous data as evidence. The observation data provided by the Lysimeter were compared with the average ETa provided by the Luancheng Station 3 × 3 around a 1 km pixel to reduce the interference of the ambient uncertainties.

**Figure 4:**
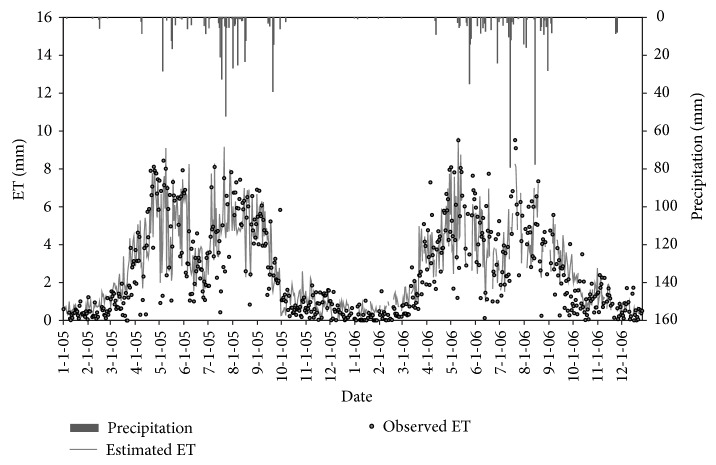
The rainfall (column), the model estimates Eta (line), and the Lysimeters observed Eta (point) from 2005 to 2006.

Figure 4 shows the comparison between the estimated ETa and the measured value from 2005 to 2006. It can be seen that the estimated ETa using the model is in a good agreement with the measured values. Fluctuations in the state of the two are almost the same. The data show two distinct peak times, one in 4-5 months and one in 7–9 months, because of the winter wheat-summer corn rotation. Based on the statistical analysis, the daily average deviation is 0.86 mm and the SEE is 1.2 mm between the estimation and the observed values.

### 3.2. ETa Temporal and Spatial Variations

**Figure 5:**
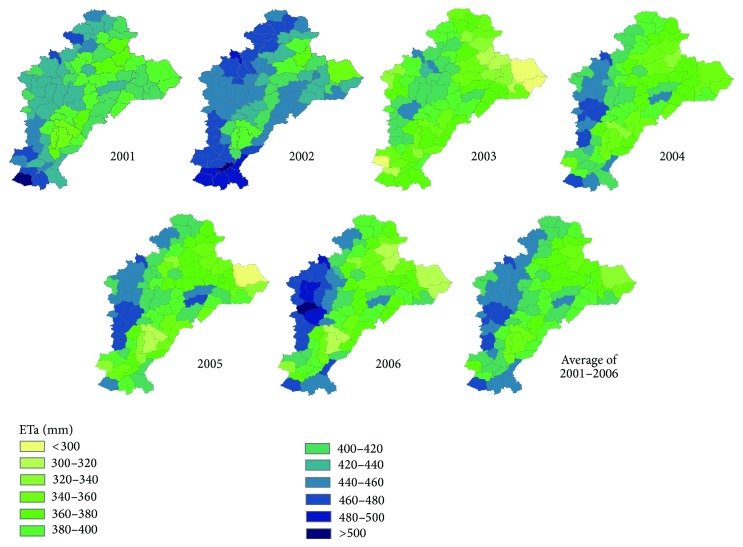
Winter wheat's ETa in Hebei Plain from 2001 to 2006.

Figure 5 shows the ETa spatial distribution of the Hebei Plain 2001–2006 winter wheat growing season (October 5 to June 10 of the following year). The figure shows that the annual ETa is greater in the western than the eastern regions of the study area. Because the western region is one of the major winter wheat-producing areas, its soil and climate are more appropriate; in addition, the developed irrigation systems ensure the moisture requirement for the growth of winter wheat. In contrast, in the eastern region, particularly in the northeast (top right), the winter wheat growing fields are mostly dependent on the rain. Thus, the ETa is much lower. The reason why the ETa is relatively low in the southeast area (lower right) is also the same as that of the northeast area.

In Table 1, the average, standard deviation, and maximum and minimum values are listed for the annual ETa in the research area. From the table, we can see that the ETa denotes a minimum of 372.1 mm in 2003 and a maximum of 446 mm in 2002 and is between 390 mm and 420 mm in the rest of those years, where the ETa in 2001 is higher than the other years and in the rest of the years is approximately 400 mm.

The highest total rainfall during the wheat growing season reached 152.8 mm in 2003, the amount representative of the wet year in the case of 50 years (1957–2006) of rainfall probability. The minimum rainfall happened in 2006 reaching only 88.1 mm and was followed by 2005 which was 105.7 mm. Both of these years were water shortage years. The rainfall amount in 2001 and 2002 was 126.3 mm and 124.4 mm, respectively. Thus, those two years are normal rainfall years. However, in 2003, the rainfall was up to 248.7 mm because the rainfall was above normal in the fall. During the period from October 10 to October 12, the strongest rainfall occurred which represents the highest amount in nearly 50 years of the same period in the history. However, ETa was not the highest in 2004. According to the 2001–2006 growing season rainfall and the ETa comparative analysis, we can see that rainfall has little effect on the ETa in the area, which is the main reason that the extensive irrigation system supplements the rainfall decline, such as for the ETa in 2006. Relative to the rainfall, the temperatures have a greater impact on the ETa during the growing season. From Table 2, both the ETa and the temperature were the lowest in 2003, which also belonged to the low-temperature year. At the same time, both the ETa and the temperature were the highest in 2002.

For the 480 points of county-level data (80 counties and 6 years), the ETa accounted for 38.3% (up to 184 points) of the total range from 400 mm to 450 mm and for 73.1% of the total range from 350 mm to 450 mm. The result is consistent with the result obtained using the models and the remote sensing data [12, 16–20]. The result is also in accordance with the ETa range of the winter wheat growing season under the conditions of full irrigation determined by field experiments [11, 18, 28–34].

### 3.3. CWP Temporal and Spatial Variations

**Figure 6:**
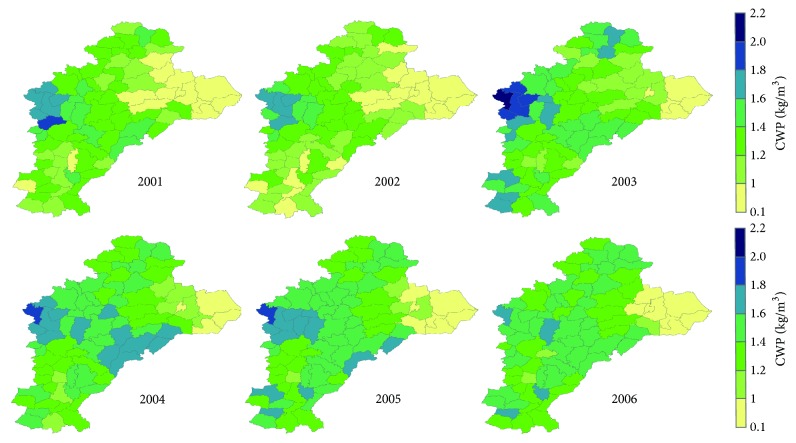
CWP of study area from 2001 to 2006.

Figure 6 represents the average CWP distribution from 2001 to 2006. According to Figure 6, the CWP in the western and central areas was higher than that in the other regions, ranging from 1.2 kg/m^3^ to 2.2 kg/m^3^.

In particular, in the central west region, the CWP is the highest, ranging from 1.6 kg/m^3^ to 2.2 kg/m^3^. In the eastern region, the CWP is mostly lower than 1 kg/m^3^. An average of 1.32 kg/m^3^, a maximum of 2.23 kg/m^3^, and a minimum value of 0.05 kg/m^3^ were found in the 480 CWP points of the research area (80 counties, 6 years). After removal of less than 5% and greater than 95% of the cumulative frequency distribution, the frequency distribution ranges from 0.29 to 1.67 kg/m^3^. The other findings of the region were 0.12–2.15 kg/m^3^ [15], 0.05–1.58 kg/m^3^ [16], 0.2–1.63 kg/m^3^ [29], and 0.66–1.06 [30–34].

Table 2 lists the CWP in different years: it was lower in 2001 and 2002, showed little increase from 2003 to 2006, and reached the highest value in 2004. The standard deviation of CWP was the highest in 2003 and the lowest in 2004. The data also indicated that the spatial variability was the highest in 2003, while it was the lowest in 2004.

**Figure 7:**
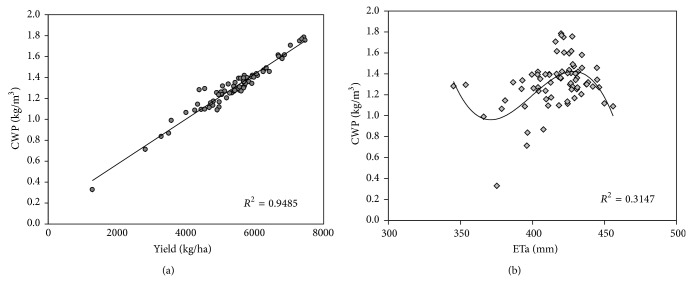
The relationship between yield and CWP (a) and the relationship between ETa and CWP (b).

Through studying the relationship between the yield and the ETa with the average CWP in many years, it can be concluded that the CWP was positively correlated with the yield, with an *R* ^2^ of 0.9, as shown in Figure 7. Although the relationship was more complex between the CWP and the ETa, it was nearly a cosine curve. In the eastern region, the ETa was low, while the decrease of yields of the regions was lower than the decrease of ETa. Thus, CWP was maintained at a relatively high level. In contrast, both the ETa and yield increased, whereas the CWP declined. In the middle region, the magnitude of the increasing yield was greater than the ETa and the CWP also increased. When the range of the ETa was 410 to 430 mm, the CWP was maximized. With a further increase in the ETa, the yield began to lower the rate of increase and the CWP also declined.

## 4. Conclusion

To achieve long-term sustainable agricultural production and to manage agricultural water, it is essential to fully understand the spatial and temporal variations of the CWP and crop water consumption. The crop water consumption, the CWP, and its temporal changes and influencing factors were estimated using the energy balance-ET model (EBEM) based on the remote sensing data from the northern Hebei Plain areas of the NCP. The results indicated that the EBEM model can use the MODIS data to provide a good estimate of the daily net radiation and daily evapotranspiration. The average ratio of 0.976, the relative error of 6.7%, and the SEE of 0.82 MJ m^−2^ day^−1^ were between the estimated and measured value of the *R*_*n*_. The daily average deviation of 0.86 mm and the SEE of 1.2 mm were between the ETa estimates and measurements obtained using the Lysimeter.

According to the ETa and CWP spatial analysis of the winter wheat growing season, the ETa and CWP in the west were all higher than in the east from 2001 to 2006. In the study area, the ETa was mainly distributed in the range of 350 to 450 mm and the CWP was mainly distributed in the range of 0.29 to 1.67 kg/m^3^ during the wheat growing season. Because the irrigation system is highly developed in the western region, it satisfied the requirements for generating the ETa. However, the heat condition required for the ETa is dependent on the interannual temperature fluctuations. Although the CWP was affected by the yield and the water consumption, it is significantly positively correlated with the yield, and complex fluctuation relationships occurred between the ETa and the CWP.

## Figures and Tables

**Table 1:** The average, standard deviation, maximum and minimum values of ETa in all 80 counties from 2001 to 2006 (mm).

Seasons	Mean	SD	Max.	Min.
2001	417.1	29.8	509.9	345.5
2002	446.0	29.9	506.1	356.3
2003	372.1	37.3	433.3	261.3
2004	392.9	32.0	456.2	336.6
2005	389.9	38.3	465.5	289.9
2006	400.1	49.1	516.3	303.7

**Table 2:** The average, standard deviation, maximum and minimum values of CWP in all 80 counties from 2001 to 2006 (kg m^−3^).

Seasons	Mean	SD	Max.	Min.
2001	1.24	0.27	1.81	0.43
2002	1.15	0.27	1.68	0.05
2003	1.51	0.30	2.18	0.22
2004	1.33	0.21	1.78	0.60
2005	1.22	0.24	1.72	0.38
2006	1.26	0.24	1.68	0.23
